# Comparison of Transfer Learning and Conventional Machine Learning Applied to Structural Brain MRI for the Early Diagnosis and Prognosis of Alzheimer's Disease

**DOI:** 10.3389/fneur.2020.576194

**Published:** 2020-11-05

**Authors:** Loris Nanni, Matteo Interlenghi, Sheryl Brahnam, Christian Salvatore, Sergio Papa, Raffaello Nemni, Isabella Castiglioni, The Alzheimer's Disease Neuroimaging Initiative

**Affiliations:** ^1^Department of Information Engineering, University of Padua, Padua, Italy; ^2^Institute of Molecular Bioimaging and Physiology, National Research Council of Italy (IBFM-CNR), Milan, Italy; ^3^Department of IT and Cybersecurity, Missouri State University, Springfield, MO, United States; ^4^Department of Science, Technology and Society, Scuola Universitaria Superiore IUSS Pavia, Pavia, Italy; ^5^DeepTrace Technologies S.R.L., Milan, Italy; ^6^Centro Diagnostico Italiano S.p.A., Milan, Italy; ^7^Department of Physics “G. Occhialini”, University of Milano Bicocca, Milan, Italy

**Keywords:** artificial intelligence, deep learning, magnetic resonance imaging, Alzheimer's disease, mild cognitive impairment, transfer learning, CNN–convolutional neural networks

## Abstract

Alzheimer's Disease (AD) is the most common neurodegenerative disease, with 10% prevalence in the elder population. Conventional Machine Learning (ML) was proven effective in supporting the diagnosis of AD, while very few studies investigated the performance of deep learning and transfer learning in this complex task. In this paper, we evaluated the potential of ensemble transfer-learning techniques, pretrained on generic images and then transferred to structural brain MRI, for the early diagnosis and prognosis of AD, with respect to a fusion of conventional-ML approaches based on Support Vector Machine directly applied to structural brain MRI. Specifically, more than 600 subjects were obtained from the ADNI repository, including AD, Mild Cognitive Impaired converting to AD (MCIc), Mild Cognitive Impaired not converting to AD (MCInc), and cognitively-normal (CN) subjects. We used T1-weighted cerebral-MRI studies to train: (1) an ensemble of five transfer-learning architectures pretrained on generic images; (2) a 3D Convolutional Neutral Network (CNN) trained from scratch on MRI volumes; and (3) a fusion of two conventional-ML classifiers derived from different feature extraction/selection techniques coupled to SVM. The AD-vs-CN, MCIc-vs-CN, MCIc-vs-MCInc comparisons were investigated. The ensemble transfer-learning approach was able to effectively discriminate AD from CN with 90.2% AUC, MCIc from CN with 83.2% AUC, and MCIc from MCInc with 70.6% AUC, showing comparable or slightly lower results with the fusion of conventional-ML systems (AD from CN with 93.1% AUC, MCIc from CN with 89.6% AUC, and MCIc from MCInc with AUC in the range of 69.1–73.3%). The deep-learning network trained from scratch obtained lower performance than either the fusion of conventional-ML systems and the ensemble transfer-learning, due to the limited sample of images used for training. These results open new prospective on the use of transfer learning combined with neuroimages for the automatic early diagnosis and prognosis of AD, even if pretrained on generic images.

## Introduction

With an estimate of 5.7 million people affected in 2018 in the only United States and a prevalence of 10% in the elder population [> 65 years old, (1)], Alzheimer's Disease (AD) is the most common neurodegenerative disease, accounting for 50–75% of all cases of dementia (2).

To date, AD can be definitely diagnosed only after death, with post-mortem examinations aimed at measuring the presence of amyloid plaques and neurofibrillary tangles. Distinguishing between different neurodegenerative phenotypes of dementia is of paramount importance to allow patients accessing appropriate treatment and support (3).

A probable or possible clinical diagnosis of AD is often mainly based on patient's self-reported experiences and the assessment of behavioral, functional, and cognitive status through neuropsychological tests and questionnaires. However, this approach results to be insufficient for the diagnosis of AD, especially in the early pre-dementia stage of the disease known as Mild Cognitive Impairment (MCI), whose rate of progression to Alzheimer's dementia is only 33% (4).

Due to these weaknesses and according to many scientific evidences arising in the last years, the revised diagnostic criteria for AD published in 2011 included neuroimaging studies as techniques able to detect signs of the disease even before dementia is apparent (5, 6). Neuroimaging techniques include both functional imaging, such as Positron Emission Tomography (PET) with Ab- or tau-specific radiotracers, and structural/metabolic imaging, such as Magnetic Resonance Imaging (MRI) or PET with Fluoro-deoxiglucose radiotracer. These methods can provide measurements of AD-specific proteins' deposit and reduced metabolism/atrophic regions, respectively, related to the presence and the progression of AD (7, 8).

Scientific progress led to a more recent initiative by the National Institute on Aging and Alzheimer's Association to update the 2011 guidelines, defining AD by its underlying pathologic processes that can be documented by postmortem examination or *in vivo* by biomarkers, irrespectively from the clinical symptoms or signs. This new approach shifted the definition of AD in living people from a syndromal to a biological construct and focused the diagnosis of AD on biomarkers in living persons by means of the measure of β amyloid deposition, pathologic tau, and neurodegeneration from biofluids and imaging (9).

However, it can be difficult for radiologists to detect the presence of imaging biomarkers by visual inspection of brain images at early disease stages.

Because of these limitations, the neuroimaging community has recently been attracted by advanced machine-learning (ML) and pattern-recognition techniques. These techniques are indeed able to extract information from data and to use this information to design models that can automatically classify new samples. In the field of neuroimaging, they proved able to identify unknown disease-related patterns in imaging data without a-priori information about the pathophysiological mechanisms of the underlying disease. The performance of these techniques in automatically diagnosing new patients reached high values, also when considering AD [e.g., (10–14)]. However, in the early diagnosis of AD, i.e., the discrimination of MCI patients who will convert to AD (MCIc) from those who will not (MCInc) is a very complex and challenging issue (15) since the clinical implementation of ML systems trained on subtle brain features able to discriminate MCIc from MCInc requires the development of a large variety of parameters and image processing methods for the fine tuning of the training.

In the very last years, a new ML technique from the computer-vision field came to the attention of the research community because of the excellent results obtained in several visual recognition tasks (16). This technique, known as deep learning (17), allows learning representations of data with multiple levels of abstraction (represented by multiple processing layers) (18), which result in an astounding improvement in terms of performance with respect to conventional classification algorithms.

Deep learning has already shown its potential performance in clinical applications, such as the automatic detection of metastatic breast cancer (19), the automatic lung-cancer diagnosis (20), or the automatic segmentation of liver tumor (21), both by training a whole classification architecture from scratch or by using transfer-learning techniques (22, 23). Specifically, this last approach allows pre-training a network on a very large dataset of generic images and -then- fine tuning the resulting model using specific samples related to the target problem. This turns out to be really useful when the number of available training samples is small with respect to the number of samples required to train from scratch a stable, unbiased and not-overfitted deep-learning architecture.

The application of these emerging techniques to neuroimaging studies is an active research field for their expectations on improving classification performance. However, some criticisms about this approach exists, such as; (1) the nature of the features used as a representation of the input samples, which still need further investigation, especially when considering neurodegenerative diseases such as AD, which show a distributed (not localized) pattern of atrophy; (2) the use of transfer-learning architectures, which are often pre-trained on generic images, given the relatively small amount of medical-imaging data; however, this may affect the performance of the classification model, since the relation between the information used to pre-train an architecture and that used for fine tuning may impact the performance of the network.

Given these open issues, and the lack of scientific papers aimed at a direct comparison of the different methodological approaches, in this paper we want to focus on the comparison of deep /transfer-learning and conventional machine learning when applied to the same neuroimaging studies for the early diagnosis and prognosis of AD. Magnetic Resonance Imaging (MRI) has several points of strength for this kind of study: it is included as neuroimaging modality in all clinical trials focused on AD, to exclude patients with brain diseases different from AD; it is less expensive than PET and more widespread in both western and non-western regions; it is a non-invasive technique, and it can provide information about neuronal degeneration at a morphological level, thus serving as imaging modality for measuring biomarkers of neurodegeneration.

With this aim, we implemented and trained different classification approaches, based on deep-learning techniques and on conventional ML using two different sets of multicenter MRI brain images obtained from the Alzheimer's Disease Neuroimaging Initiative (ADNI) database (adni.loni.usc.edu). The following binary comparisons were evaluated: AD vs. CN, MCIc vs. CN, MCIc vs. MCInc. However, since individual algorithms may perform better than the others for a given task, an ensemble of different individual algorithms was implemented in this work to reduce issues across the different AD phenotype comparisons arising from the choice of a single architecture and improving the classification performances.

The automatic-classification performance of the proposed methods was used to compare the different classification approaches and to evaluate the potential application of ensemble transfer learning for the automatic early diagnosis and prognosis of AD against well-established, validated ML techniques.

## Materials and Methods

### Participants and Datasets

Data used in the preparation of this article were obtained from the Alzheimer's Disease Neuroimaging Initiative (ADNI) database (adni.loni.usc.edu). The ADNI was launched in 2003 by the National Institute on Aging (NIA), the National Institute of Biomedical Imaging and Bioengineering (NIBIB), and the Food and Drug Administration (FDA), as a 5-year public private partnership, led by the principal investigator, Michael W. Weiner, MD. The primary goal of ADNI was to test whether serial magnetic resonance imaging (MRI), positron emission tomography (PET), other biological markers, and clinical and neuropsychological assessments subjected to participants could be combined to measure the progression of mild cognitive impairment (MCI) and early Alzheimer's disease (AD) - see www.adni-info.org.

As per ADNI protocol (http://www.adni-info.org/Scientists/ADNIStudyProcedures.html), each participant was willing, spoke either English or Spanish, was able to perform all test procedures described in the protocol and had a study partner able to provide an independent evaluation of functioning. Inclusion criteria for different diagnostic classes of patients are stated below:

#### CN Subjects

Mini Mental State Examination (MMSE) (24) scores between 24 and 30, Clinical Dementia Rating (CDR) of zero (25), and absence of depression, MCI and dementia.

#### MCI Patients

MMSE scores between 24 and 30, CDR of 0.5, objective memory loss measured by education-adjusted scores on the Logical Memory II subtest of the Wechsler Memory Scale (26), absence of significant levels of impairment in other cognitive domains, and absence of dementia.

#### AD Patients

MMSE scores between 20 and 26, CDR of 0.5 or 1.0, and criteria for probable AD as defined by the National Institute of Neurological and Communicative Disorders and Stroke (NINCDS) e by the Alzheimer's Disease and Related Disorders Association (ADRDA) (27, 28).

In the present work, we used two different sets of data obtained from the ADNI repository. These replicate the same sets of data used in previously published studies (11, 29) and were referred to as *Salvatore-509* and *Moradi-264*, are described in detail below.

#### Salvatore-509

This dataset is the same used in a previously-published study by Salvatore et al. (12), and is composed of 509 subjects collected from 41 different radiology centers and divided as follows: 137 AD, 162 CN, 76 MCIc, and 134 MCInc, depending on their diagnosis (converted to AD or stable MCI and CN) after a follow-up period of 18 months For each patient, T1-weighted structural MR images (1.5 T, MP-RAGE sequence) acquired during the screening or the baseline visit were considered [according to the standard ADNI acquisition protocol detailed in (30)]. Data were obtained from the ADNI public repository.

#### Moradi-264

This is the same dataset used in a previously-published study by Moradi et al. (11), and is composed of 264 subjects divided into 164 MCIc and 100 MCInc depending on their diagnosis (converted to AD or stable MCI) after a follow-up period of 36 months. For each patient, T1-weighted structural MR images (1.5 T, MP-RAGE sequence) acquired during the baseline visit were considered. In the original publication, the binary classification of MCIc vs. MCInc was explored and the classification system was validated through a 10-fold CV approach. Also in this case the data were obtained from the ADNI public repository.

### MRI Preprocessing

For both *Salvatore-509* and *Moradi-264* datasets, MR images were downloaded in 3D NIfTI format from the ADNI repository. Image features (e.g., resolution) were not the same for each scan in the datasets, because MRIs were obtained from a multicenter study. Each image was then further subjected to a preprocessing phase, individually, with the main aim to make different scans comparable to each other. This phase was entirely performed on the Matlab platform (Matlab R2017a, The MathWorks) using the VMB8 software package. The preprocessing phase consisted in image re-orientation, cropping, skull-stripping, co-registration to the Montreal Neurological Institute (MNI) brain template, and segmentation into gray matter tissue probability maps. Specifically, the co-registration step was performed using the MNI152 (T1 1 mm brain) (31). Possible inhomogeneities and artifacts were checked by visual inspection on MRI volumes before and after the pre-processing step.

All entire volumes of MRI resulted to be of size 121 x 145 x 121 voxels.

The MRI volumes were then cropped to the 100 central slices, in order to focus the analysis to the inner brain structures (including hippocampus), thus resulting in a 100 x 100 x 100-voxels volume for each MRI scan.

In the subsequent analyses, both the entire MRI volumes and the inner cerebral structures were used, separately.

### Classification Algorithms

In this section, we describe the conventional-ML and deep/transfer-learning approaches that were used to perform automatic classification of AD diagnosis and prognosis.

We designed 1 Support-Vector-Machine classifier [SVM, (32)] coupled to two different feature extraction/selection techniques, five fine-tuned 2D Convolutional Neural Networks [CNNs, (17)] pretrained on generic images (transfer learning), and one 3D CNN trained from scratch on MRI volumes. An exhaustive description of the different approaches used in this study is presented below. It is worth noting here that the choice of the conventional-ML approaches as well as of the feature-extraction-and-selection techniques used in this study come from a review of the literature (13) as well as from the results of previous research studies (33, 34).

#### Conventional-ML Approach

The conventional-ML approach used in this paper is obtained by coupling two feature extraction and/or selection technique with an automatic-classification technique based on SVM.

Given the high dimensionality of the feature vector obtained from each MR image (> 10^6^ voxels per MR volume), the feature-extraction/selection step is indeed necessary to reduce the curse-of-dimensionality issue, to remove irrelevant features, and to reduce overfitting, thus potentially improving the performance of SVM. The following feature extraction and selection approaches were tested: Aggregate Selection and Kernel Partial Least Squares. The choice of these techniques is based on the results obtained in our two previous works (33, 34), in which we studied and compared more than 30 different feature-reduction approaches (considering both papers) in order to study their discrimination power when applied to neuroimaging MRI data. As it can be drawn from the results and discussions of these papers, Aggregate Selection and kPLS show the most promising results in terms of classification accuracy, sensitivity, specificity, and AUC, thus showing higher discrimination power than the other tested approaches (considered individually). Accordingly, we applied these feature-reduction techniques for the classification of MRI data in this work.

Aggregate Selection (AS) (35) is a feature-selection technique that combines (1) the feature ranking based on the Fisher's score (32), (2) the two-sample T-test (32), and (3) the sparse multinomial logistic regression via bayesian L1 regularization (36). As these criteria quantify different characteristics of the data, considering the ranking of all the above criteria would produce a more informative set of features for classification, that is, the resulting set of features should prove superior with respect to each criterion considered individually. In practice, it is difficult to combine the ranking of all these criteria because the range of statistics is different: therefore, a criterion that generates a higher range of statistics would dominate those with a lower range. To avoid this problem, AS uses a modified analytic hierarchy process that assembles an elite set of features through a systematic hierarchy. This is accomplished by comparing the ranking features of a set of criteria by first constructing a comparison matrix whose elements are required to be transitive and consistent. Consistency of the comparison matrix is calculated using the Consistency Index (CI) and the Consistency Ratio (CR) based on large samples of random matrices. Let $\epsilon ={\left[\epsilon_{1},\epsilon_{2},\ldots ,\epsilon_{n}\right]}^{T}$ be an eigenvector and λ be an eigenvalue of the square matrix X. We would then have what follows:

(1) $$\begin{array}{r} X\epsilon =\lambda \epsilon \end{array}$$

(2) $$\begin{array}{r} CI=\frac{\lambda_{max}-n}{n-1} \end{array}$$

(3) $$\begin{array}{r} CR=CI/index \end{array}$$

If the set of judgments is consistent, CR will not exceed 0.1. If CR = 0, then the judgments are perfectly consistent. After the comparison matrices are constructed, the eigenvectors (one for each criterion) that demonstrate the ranking scores are calculated using a hierarchical analysis. This results in a *performance matrix*. The feature ranking is then obtained by multiplying the performance matrix with the vector representing the importance weight of each criterion. The weight vector is typically obtained by evaluating the level of importance of each criterion with respect to a specific aspect. In this case, the weight vector was set *a priori* to 1/(number of criteria) (1/3 in this case) in order to avoid bias.

Kernel Partial Least Squares (kPLS), first described in (37), is a feature-extraction technique that computes nonlinear correlations among the features by approximating a given matrix to a vector of labels. kPLS is a nonlinear extension of Partial Least Square (PLS), which can be viewed as a more powerful version of the Principal Component Analysis (38), as also in this case a set of orthogonal vectors (called components) is computed by maximizing the covariance between the D (observed variables or data) and C (classes or diagnostic labels).

In this paper, we used the approach proposed in the paper by Sun et al. (39), for which the entire kPLS algorithm can be stated in terms of the dot products between pairs of inputs and a substitute kernel function *K*(·, ·). If *X* ∈ *R*^*N* × *D*^ is the matrix of D-dimensional observed variables (D) and N number of observations, and if *Y* ∈ *R*^*N* × *C*^ is the corresponding matrix of C-dimensional classes (C), then we can map a nonlinear transformation Φ(·) of the data into a higher-dimensional kernel space *K*, such that $\Phi :x_{I}\epsilon R^{D}\to \Phi \left(x_{I}\right)\epsilon K$.

The first component for kPLS can be determined as the eigenvector of the following square kernel matrix for $\beta^{\Phi }:\beta^{\Phi }\lambda =K_{X}K_{y}\beta^{\Phi }$, where *K*_*X*_ is an element of the *Gram Matrix* *K*_*X*_ in the feature space, and λ is an eigenvalue. The size of the kernel matrix *K*_*X*_*K*_*y*_ is *N* × *N* regardless of the number of variables in the original matrices *X* and *Y*.

If *T* = {*t*_1_, *t*_2_, …, *t*_*h*_} is a set of components, with *h* the desired number of components, then the accumulation of variation explanation of *T* to *Y* can be written as:

(4) $$\begin{array}{r} w_{i}=\sqrt{D\frac{\sum_{l=1}^{h}\Psi \left(Y,t_{l}\right)v_{il}^{2}}{\sum_{l=1}^{h}\Psi \left(Y,t_{l}\right)}},i\in \left\{1,2,\ldots ,D\right\}, \end{array}$$

where *v*_*il*_ is the weight of the *i*^th^ feature for the *l*th component, Ψ(·, ·) is a correlation function, and Ψ(*y*_*j*_, *t*_*l*_) is the correlation between *t*_*l*_ and *Y*. Larger values of *w*_*i*_ represent more explanatory power of the *i*^th^ feature to *Y*.

In kernel space, kPLS becomes an optimization problem:

(5) $$\begin{array}{r} \underset{\alpha \subseteq R^{N}}{argmax}\left\{\alpha^{T}S_{1}^{\Phi }\frac{\alpha }{\alpha^{T}}S_{2}^{\Phi }\alpha \right\} \end{array}$$

where α is an appropriate projection vector, and $S_{1}^{\Phi }$ and $S_{2}^{\Phi }$ are the inter-class scatter matrix and intra-class scatter matrix, respectively. The calculation of the contribution of the *l*th component γ_*l*_ can be calculated as:

(6) $$\begin{array}{r} \gamma_{l}=\frac{\sum_{i=1}^{C}N_{i}m_{il}^{\Phi }}{\sum_{i=1}^{C}N_{i}} \end{array}$$

where *N*_*i*_ is the number of samples in the *i*th class, and $m_{il}^{\Phi }$ is the mean vector of the *i*th class with respect to the *l*th component in the projection space. The larger γ_*l*_, the more significant the classification.

In this paper, we implemented the same version of kPLS proposed in the work by Sun et al. (39) and available at https://github.com/sqsun/kernelPLS. The number of components was set to 10.

The features extracted and/or selected from the original MRI volumes using these two different approaches were then used as input to the SVM classifier of the LibSVM library in Matlab (40) to perform the classification tasks.

The two SVM models obtained from the first phase of this study were then used as a fusion of conventional-ML classifiers.

#### Transfer-Learning Approach

The transfer-learning approach used in this paper is based on an ensemble of CNNs pretrained on generic images. CNNs are a class of deep feed-forward artificial neural networks (ANNs) that are shift and scale invariant. As with ANNs, CNNs are composed of interconnected neurons that have learnable weights and biases. Each of the neurons has inputs and performs a dot product, optionally followed by a non-linearity.

CNN layers have neurons arranged in three dimensions: width, height and depth. In other words, each layer in a CNN transforms a 3D input volume into a 3D output volume of neuron activations. CNNs are the repeated concatenation of five different classes of layers: convolutional, activation, pooling, fully-connected, and classification layers.

The convolutional layers make up the core building blocks of a CNN and are the most computationally expensive. They compute the outputs of neurons that are connected to local regions by applying a convolution operation to the input. The receptive field is a hyperparameter that determines the spatial extent of connectivity. A parameter sharing scheme is used in convolutional layers to control the number of parameters. The parameters of convolutional layers are shared sets of weights (commonly referred to as kernels or filters) that have a small receptive field.

Pooling layers perform a non-linear down-sampling operation. Different non-linear functions are used when implementing pooling, with max pooling being one of the most common methods. Max-pooling partitions the input into a set of non-overlapping rectangles and outputs the maximum for each group. Thus, pooling reduces the spatial size of the representation, reducing the number of parameters and the computational complexity of the network as a consequence. The reduction of features involved results in a reduction of overfitting issues. A pooling layer is commonly inserted between convolutional layers.

Activation layers apply some activation function, such as the non-saturating function *f*(*x*) = max(0, *x*) or the saturating hyperbolic tangent *f*(*x*) = *tanh*(*x*) or the sigmoid function *f*(*x*) = (1 + *e*^−*x*^)^−1^.

Fully-connected layers have full connections to all activations in the previous layer and are applied after convolutional and pooling layers. A fully-connected layer can easily convert to a convolutional layer since both layers compute dot products, i.e., their functional form is the same.

The last layer is the classification layer, which performs the classification step and returns the output of the deep-learning network.

Given the relatively low number of data available in this study (from a minimum of 76 to a maximum of 164 patients per diagnostic class) with respect to the number of samples needed to train a CNN model that is stable, unbiased and not-overfitted, we could not train an entire deep-learning architecture from scratch (with random initialization). Training a CNN from scratch indeed requires more training samples than those necessary for training conventional ML. The number of training samples can decrease significantly if pre-trained models are used, such as in transfer learning (22, 23), in which a CNN is pretrained on a very large dataset of (usually) generic image, the weights of the pretrained network are then fine-tuned using samples that are specifically related to the target problem, and the classification layer on top of the network is replaced accordingly.

In order to reduce issues arising from the choice of a single network, we used different pretrained networks, thus designing different CNN models. The classification outputs of these architectures were then merged via sum rule in an ensemble transfer-learning approach. The following pretrained networks were used:

- AlexNet [winner of the ImageNet ILSVRS challenge in 2012; (17)] is composed of both stacked and connected layers and includes five convolutional layers followed by three fully-connected layers, with max-pooling layers in between. A rectified linear unit nonlinearity is applied to each convolutional layer along with a fully-connected layer to enable faster training.
- GoogleNet [winner of the ImageNet ILSVRS challenge in 2014; (41)] is the deep-learning algorithm whose design introduced the so-called Inception module, a subnetwork consisting of parallel convolutional filters whose outputs are concatenated. Inception greatly reduces the number of required parameters. GoogleNet is composed by 22 layers that require training (for a total of 27 layers when including the pooling layers).
- ResNet [winner of ILSVRC 2015; (42)], an architecture that is approximately twenty times deeper than AlexNet; its main novelty is the introduction of residual layers, a kind of network-in-network architecture that forms building blocks to construct the network. ResNet uses special skip connections and batch normalization, and the fully-connected layers at the end of the network are substituted by global average pooling. Instead of learning unreferenced functions, ResNet explicitly reformulates layers as learning residual functions with reference to the input layer, which makes the model smaller in size and thus easier to optimize than other architectures.
- Inception-v3 (43), a deep architecture of 48 layers able to classify images into 1,000 object categories; the net was trained on more than a million images obtained from the ImageNet database (resulting in a rich feature representation for a wide range of images). Inception-v3 classified as the first runner up for the ImageNet ILSVRC challenge in 2015.

CNNs are designed to work on RGB images (3-channels), while MRIs store information in 3-dimensional single-channel volumes. In order to exploit the potential of transfer learning through pretrained 2D architectures, we introduced a technique to decompose 3D MRIs to 2D RGB-like images that could be fed into 2D pretrained architectures for transfer-learning purposes.

In this technique, MRI slices, once co-registered to the MNI brain template, are used as 2D RGB bands. Specifically, 3 different MRI slices are stacked together to form one RGB-like image. We then introduced the variable “gap,” which corresponds to the “distance” between the 3 MRI slices (i.e., the one used for the “R” band and those used for the “G” and “B” bands). The variable “gap” is measured as the difference among the number of MRI slices (e.g., if “gap” is 1, the 3 MRI slices are adjacent).

Four different approaches were used (A, B, C, D) to form a RGB-like image, depending on the orientation of the considered MRI slices, i.e., sagittal, coronal, or transaxial:

A. “R” band is the n-th sagittal slice of the original 3D MRI volume, “G” band is the (n-th + gap) sagittal slice of the original 3D MRI volume, and “B” band is the (n-th + 2×gap) sagittal slice of the of the original 3D MRI volume;
B. “R” band is the n-th coronal slice of the original 3D MRI volume, “G” band is the (n-th + gap) coronal slice of the original 3D MRI volume, and “B” band is the (n-th + 2×gap) coronal slice of the original 3D MRI volume;
C. “R” band is the n-th transaxial slice of the original 3D MRI volume, “G” band is the (n-th + gap) transaxial slice of the original 3D MRI volume, and “B” band is the (n-th + 2×gap) transaxial slice of the original 3D MRI volume;
D. “R” is the n-th sagittal slice of the original 3D MRI volume, “G” band is the (n-th + gap) coronal slice of the original 3D MRI volume, and “B” band is the (n-th + 2×gap) transaxial slice of the original 3D MRI volume.

(Approach D is the only one in which different MRI-slice orientations are used together).

Different transfer-learning models were trained for different configurations of RGB-like images of the subjects.

Training-and-classification tests were run with gap ∈ {0,1,2} and n starting from the first slice of the original 3D MRI volume. For example, considering the approach “A,” when gap = 1 and n = 1, the corresponding RGB-like image is a 2D image in which “R,” “G,” and “B” bands are the first three sagittal slices of the original 3D MRI volume.

In this way, the intra-subject spatial structural information is preserved, both in the x-y plane and in the z dimension. In the example above, each 2D RGB-like image contains the entire original MRI information on the sagittal plane. The same is true along the sagittal direction, although limited to the depth of three slices.

Moreover, different transfer-learning models were used for each configuration of RGB-like images of the subjects.

For example, the RGB-like image of a patient centered on slice #2 is used in the learning-and-classification process only with RGB-like images of the other patients centered on the same slice #2.

As MRI volumes are co-registered to the same MNI brain template, all RGB-like images centered on the same slice also result to be co-registered among them, and thus the data used to train each transfer-learning model refer to the same MNI coordinates and contain the same morphological and structural information across subjects.

In this way, the inter-subjects spatial structural information is preserved.

As each individual model (one for each value of “n”) returned an individual classification output, the final classification was obtained by merging (via sum rule) the classification output of all the RGB-like images that compose a given MRI volume.

It must be noted that in this process the “depth” of CNN layers corresponds to the 3 different MRI slices that are used as RGB bands. The learning process by convolutional filters of CNN layers uses the spatial structural information from these 3 RGB bands. Moreover, regarding the pooling process, the maximum dimension of the pooling-filter matrix is 3 by 3, thus poorly impacting the spatial size of the image representation.

The optimal number of epochs was evaluated as the number of epochs for which the network reached the minimum training error (convergence). The learning rate was set to 0.0001, and a mini batch with 30 observations at each iteration was used. No data augmentation was performed.

The deep-learning models obtained from the first phase of this study were then used in an ensemble of transfer-learning models. All transfer-learning analyses were performed on a computing system with a dedicated Nvidia GeForce GTX Titan X GPU (12 GB memory size).

#### Deep-Learning Approach

A new 3D CNN was trained from scratch using the MRI volumes from the two considered datasets.

Our 3D CNN architecture was composed of the following 3D layers: (1) convolutional layer, (2) Rectified Linear Unit (ReLU) layer, (3) max-pooling layer, (4) fully-connected layer, (5) soft-max layer, and (6) final classification layer.

The size of the 3D input layer was set equal to 28 by 28 by 121. The stride of convolutional layers and the stride of the max-pooling layers were set equal to 4.

In order to perform the training and optimization of the network, MRI scans were then split up into patches of size 28 by 28 by 121, without overlap, and the final classification was obtained by merging (via sum rule) the classification output of all the patches that compose a given MRI volume.

Training and optimization of the network were performed using Stochastic Gradient Descent with Momentum (SGDM) optimization, initial learning rate equal to 0.0001. Analyses were performed on a computing system with a dedicated Nvidia GeForce GTX Titan X GPU (12 GB memory size).

### Performance Evaluation and Comparison of Different Methods

The following classification methods were tested: AS + SVM, kPLS + SVM, a fusion between these two SVMs (Method #1), five 2D transfer-learning architectures considered individually, i.e., AlexNet, GoogleNet, ResNet50, ResNet101, InceptionV3, one 3D CNN trained from scratch on the MRI volumes, and an ensemble between these CNNs via sum rule (Method #2).

The performance-evaluation procedure was performed -for both Salvatore-509 and Moradi-264 datasets—using (1) the same validation strategy, (2) the same validation indices, and (3) the same binary comparisons adopted in the original papers. In both cases, all the classification steps (including feature extraction/selection and the automatic classification itself) were embedded into a nested cross-validation (CV) strategy. This allowed us to perform an optimization of the input parameters, i.e., to find the best configuration of parameters to be used for this specific classification task. However, in order to reduce overfitting issues, we kept the number of hyperparameters to be optimized as low as possible.

The following binary comparisons were performed using the *Salvatore-509* dataset: AD vs. CN, MCIc vs. CN, MCIc vs. MCInc. When using the *Moradi-264* dataset, only the MCIc vs. MCInc binary comparison was performed due to the lack of AD and CN studies in this dataset. The performance of the proposed classification methods for these comparisons was evaluated by means of AUC (Area Under the ROC Curve) (44).

After evaluating the learning systems individually, Method #1 and Method #2 were trained and tested using (1) the entire MRI volume or (2) the inner cerebral structures (including the hippocampal region) derived from the entire MRI volume.

## Results

### Participants, Datasets, and MRI Preprocessing

Demographic characteristics of the patients considered in this paper are consistent with those reported by previous studies that used the same sets of data from the ADNI public repository. Specifically, regarding the *Salvatore-509 dataset*, the four groups of participants (namely AD, MCIc, MCInc and CN) did not show statistically-significant differences for age and gender, while statistically-significant differences were found for MMSE scores between CN and AD and between CN and MCIc. Regarding the *Moradi-264 dataset*, the two groups of patients (MCIc and MCInc) showed a matched age range (55-89 for MCIc, 57-89 for MCInc), but a slight predominance of males in the MCInc group (66%) with respect to the MCIc group (59%). More details can be found in the original publications (11, 12), respectively.

**Figure 1:**
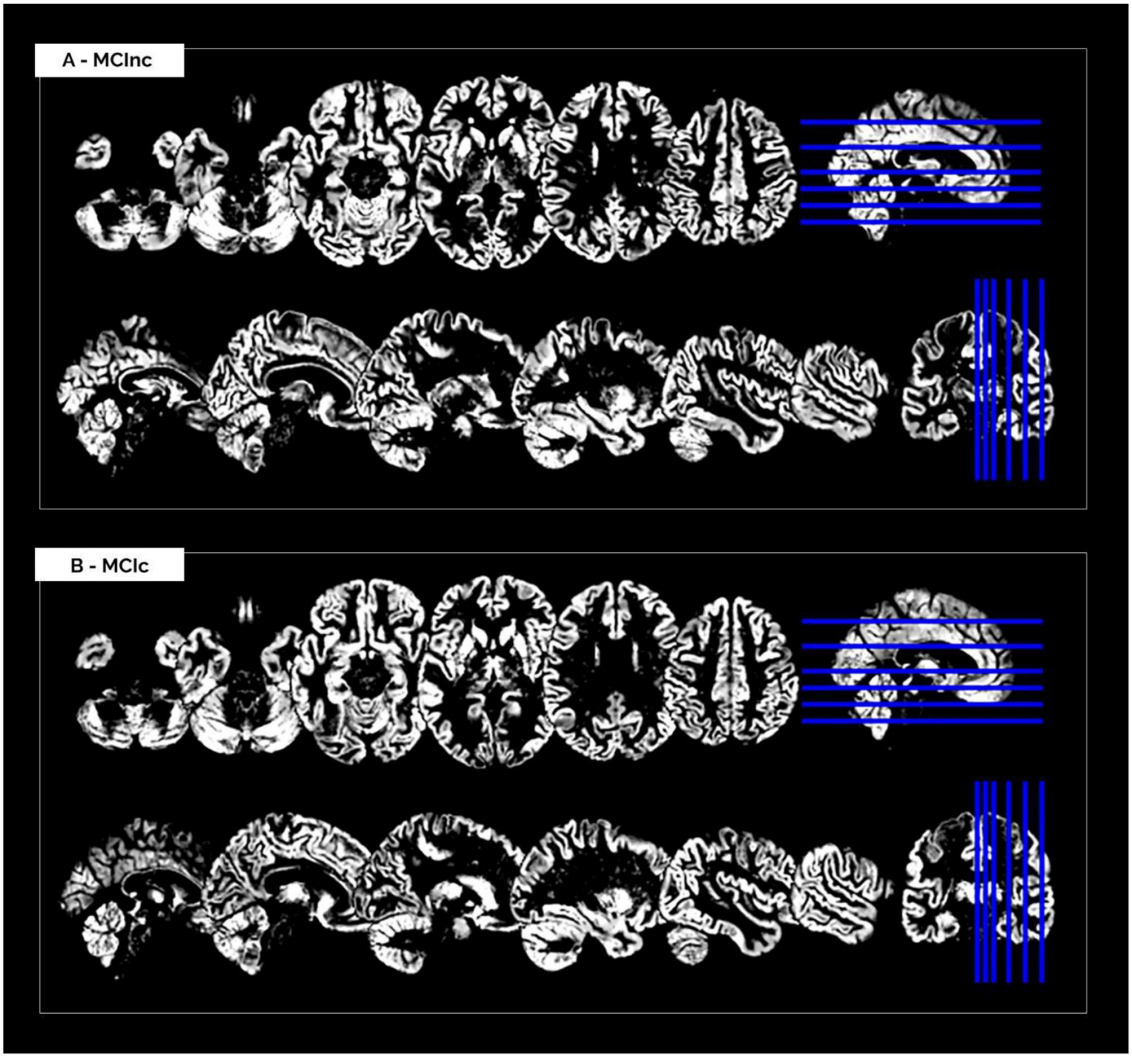
Cerebral MRI sections of an MCInc patient **(A)** and an MCIc patient **(B)** from the *Moradi-264* dataset after preprocessing (including co-registration to MNI template and segmentation into gray matter). Sections are shown in both axial (up) and sagittal (down) views. In the axial view, slices 20, 30, 40, 50, 65, and 80 are reported; in the sagittal view, slices 61, 66, 71, 80, 90, and 100 are reported.

The MRI pre-processing, including re-orientation, cropping, skull-stripping, co-registration and segmentation, was performed correctly for all the scans in the two considered datasets. Images did not show any inhomogeneities or artifacts at visual inspection, both before and after the MRI pre-processing. Figure 1 shows (as a representative example) the MRI scans of an MCInc patient (A) and an MCIc patient (B) from the *Moradi-264 dataset* after the pre-processing phase, in terms of gray matter tissue probability maps co-registered to the MNI template. Sections are shown in both axial (up) and sagittal (down) views. In the axial view, slices 20, 30, 40, 50, 65, and 80 are reported; in the sagittal view, slices 61, 66, 71, 80, 90, and 100 are reported.

### Classification, Performance Evaluation, and Comparison of Different Methods

Table 1 shows the classification performance of the considered conventional ML approaches for classifying AD vs. CN, MCIc vs. CN and MCIc vs. MCInc, respectively. The results were obtained for the two feature extraction/selection plus SVM on the entire MRI volumes. The performance obtained by the two conventional-ML classifiers in terms of AUC are comparable and accurate for both tasks AD vs. CN (>0.93) and MCIc vs. CN (>89.5), showing some limitations in the MCIc vs. MCInc task (<70%). No statistical difference was found between the performances obtained by the two methods reported in Table 1 (statistical comparison by one-way ANOVA). Accordingly, we did not choose a single SVM-based classifier for the subsequent analyses, but we performed a fusion of SVMs.

**Table 1:** Classification performance in terms of AUC of AS+SVM and kPLS+SVM.

**Conventional ML**	**AD vs. CN**	**MCIc vs. CN**	**MCIc vs. MCInc**
AS + SVM	93.1	89.6	69.1 ± 6.4^*^
kPLS + SVM	93.3	90.8	65.7 ± 3.0^*^

*Results refer to the models using the entire MRI volumes*.

* *Mean and standard deviation calculated over Salvatore-509 and Moradi-264 datasets*.

Table 2 shows the classification performance of transfer-learning architectures for classifying AD vs. CN, MCIc vs. CN and MCIc vs. MCInc, respectively, on the entire MRI volumes. The results were obtained for two pre-trained architectures (i.e., AlexNet and GoogleNet) on different configurations in terms of Gap (0, 1, 2, or combination of them via sum rule) and *MRI-decomposition* approach (A, B, C, D, or combination of them via sum rule).

**Table 2:** Classification performance in terms of AUC of AlexNet and GoogleNet after fine tuning using different Gap values and MRI-decomposition approaches.

**Pretrained architecture**	**Gap**	**MRI-decomposition approach**	**AD vs. CN**	**MCIc vs. CN**	**MCIc vs. MCInc**
AlexNet	0	A	88.2	81.9	65.5 ± 5.2^*^
	0	B	87.0	79.8	63.8 ± 1.8^*^
	0	C	89.6	74.7	61.7 ± 3.0^*^
	0	D	90.1	82.2	67.1 ± 2.3^*^
	0	A, B, C, D (combination)	90.4	83.2	67.0 ± 1.7^*^
	0, 1, 2 (combination)	A, B, C, D (combination)	90.8	84.2	69.1 ± 1.3^*^
GoogleNet	0	A	86.4	80.2	65.9 ± 0.9^*^
	0	B	87.2	75.0	68.9 ± 0^*^
	0	C	86.3	78.0	68.6 ± 0.2^*^
	0	D	87.5	79.6	67.1 ± 2.6^*^
	0	A, B, C, D (combination)	88.6	80.2	70.1 ± 0.6^*^
	0, 1, 2 (combination)	A, B, C, D (combination)	89.6	81.6	70.0 ± 1.3^*^

*Results refer to the models that were fine-tuned using the entire MRI volumes. The combination of different Gap values or MRI-decomposition approaches is simply referred to as combination*.

* *Mean and standard deviation calculated over Salvatore-509 and Moradi-264 datasets*.

The classification performance (AUC) shows that the combination of different Gaps and different *MRI-decomposition* approaches improves the power of the architecture in the discriminating tasks. We thus applied this final approach (combination of three Gaps and four *MRI-decomposition* approaches) to all transfer-learning architectures for the following analyses. This result shows that different decomposition approaches may be able to retain different spatial information, and thus the combination of different decomposition techniques may be a useful way to exploit as much spatial information as possible without having to train from scratch a new 3D network (which is much more expensive in terms of computational costs).

Table 3 shows the classification performance of all the 5 considered pre-trained architectures (entries from 1 to 5) and of the 3D CNN trained from scratch on the MRI volumes (entry n. 6) for classifying AD vs. CN, MCIc vs. CN and MCIc vs. MCInc, respectively. For pretrained 2D architectures, the results were obtained after fine tuning on the entire MRI volumes. Convergence to the minimum training error was reached for all the 2D pretrained architectures within 20 epochs. The best-performing network for both AD vs. CN and MCIc vs. CN is AlexNet, with 90.8 and 84.2% AUC, respectively. The best-performing for MCIc vs. MCInc is ResNet101, with mean AUC of 71.2% (averaged between the results obtained on *Salvatore-509* and *Moradi-264* datasets, see Supplementary Materials for non-averaged results), showing the similar limitations as conventional ML in the MCIc vs. MCInc task (AUC < 71.5%). However, no statistical difference was found among the 5 pretrained 2D architectures (one-way ANOVA). According to these results, we used an ensemble of such 5 architectures for the subsequent analyses.

**Table 3:** Classification performance in terms of AUC of the different deep/transfer-learning pretrained architectures considered individually.

**Architecture**	**AD vs. CN**	**MCIc vs. CN**	**MCIc vs. MCInc**
AlexNet^P^	90.8	84.2	69.1 ± 1.3^*^
GoogleNet^P^	89.6	81.6	70.0 ± 1.3^*^
ResNet50^P^	89.8	81.8	70.4 ± 1.0^*^
ResNet101^P^	89.9	82.2	71.2 ± 1.2^*^
InceptionV3^P^	88.8	79.9	69.8 ± 3.5^*^
3D CNN	84.1	72.3	61.1 ± 1.0^*^

*For pretrained 2D architectures (entries from 1 to 5), results refer to the ensemble of models trained using different Gap values (0, 1, 2) and MRI-decomposition approaches (A, B, C, D) on the entire MRI volumes*.

*P, pretrained*;

* *mean and standard deviation calculated over Salvatore-509 and Moradi-264 datasets*.

The 3D CNN reached convergence within 80 epochs with an AUC of 84.1% in classifying AD vs. CN, 72.3 for MCIc vs. CN and 61.1 for MCIc vs. MCInc, thus lower than the one obtained by both conventional ML and 2D transfer learning, due to the limited sample of images used for training. Based on these results, only the pretrained 2D architectures were used for the following analysis.

The comparison of the fusion of SVMs, built from AS+SVM and kPLS+SVM (Method #1), and the transfer-learning model, built as an ensemble of the individual pretrained architectures obtained above (Method #2), is shown in Table 4, on the entire MRI volumes or on the inner cerebral structures.

**Table 4:** Classification performance in terms of AUC of fusion of conventional ML (Method #1) and ensemble transfer learning (Method #2) using the entire MRI volumes or the inner cerebral structures (including the hippocampal region).

		**AD vs. CN**	**MCIc vs. CN**	**MCIc vs. MCInc**
Method #1: Fusion of 2 SVMs	Entire MRI volume	93.2	90.6	69.1 ± 6.9^*^
	Inner cerebral structures (including the hippocampal region)	93.0	90.4	73.3 ± 0.7^*^
Method #2: Ensemble of 5 Transfer-learning models	Entire MRI volume	90.2	83.2	70.6 ± 0.1^*^
	Inner cerebral structures (including the hippocampal region)	90.4	83.0	70.6 ± 0.4^*^

* *Mean and standard deviation calculated over Salvatore-509 and Moradi-264 datasets*.

As shown in the table, the fusion of conventional-ML classifiers (method #1) seems to perform better than the ensemble transfer-learning method adopted in this work (method #2).

This is slightly true when the entire MRI volume is used and it is better appreciated when only the inner cerebral structures of the MRI volume are used. Indeed, when considering the automatic discrimination of AD vs. CN and MCIc vs. CN based on the entire MRI volume, conventional-ML classifiers obtained an AUC > 93% and >90.5, respectively, while the performance obtained by the ensemble transfer-learning classifier were 90.2 and 83.2%, respectively. However, when considering the MCIc-vs-MCInc discrimination, the performance of fused conventional MLs improve from 69.1% up to 73.3% using the inner cerebral structures instead of the entire MRI volume, while those obtained from the inner structures by the ensemble transfer-learning remain stable at 70.6%.

## Discussion

Many studies in literature evaluated the potential of conventional ML in automatically classifying AD vs. CN and MCIc vs. MCInc using only structural brain-MRI data [e.g., (11, 12, 33, 45–58)], obtaining a classification performance higher than 0.80 for the AD vs. CN task and ranging from 0.50 to 0.70 for the MCIc vs. MCInc task. Overall, conventional ML algorithms applied to neuroimaging data show a mean percentage AUC in discriminating AD vs. CN of 0.94), (as resulting from a review by 10). However, when considering the most clinically relevant comparison, MCIc vs. MCInc, the mean percentage AUC decreases until to 0.70 ± 5 (mean accuracy = 0.66 ± 0.13) (13).

With the purpose to compare deep/transfer learning and conventional ML methods on the same dataset of brain-MRI data, in the present study, we implemented and assessed different deep/transfer-learning methods for the automatic early diagnosis and prognosis of AD, including very popular pre-trained systems and training from scratch a new deep learning method. We then compared their performances with different conventional ML methods implemented and assessed for the same diagnostic and prognostic task.

Focusing on conventional ML implemented in this work, the performance obtained by the fusion of 2 conventional-SVMs classifiers (obtained from two different strategies of features extraction/selection) are comparable and accurate for both tasks of AD vs. CN (AUC > 93%) and MCIc vs. CN (AUC > 89%), showing some limitations in the more complex and challenging tasks of MCIc vs. MCInc task (AUC ~69%).

Previous studies using conventional-ML techniques on the same dataset adopted in this work, obtained comparable performance with our implemented ML methods. Specifically, in the paper by Nanni et al. (33), the authors used the *Salvatore-509* dataset obtaining 93% AUC in discriminating AD from CN, 89% for MCIc vs. CN, and 69% for MCIc vs. MCInc.

However, when a fusion strategy was applied for SVMs trained on the entire MRI scans (method #1) no effective improvement was observed in the three discriminating tasks, obtaining 93% AUC in AD vs. CN, 91% AUC in MCIc vs. CN and 69% AUC in MCIc vs. MCInc.

Regarding transfer-learning, we implemented 5 popular architectures: AlexNet, GoogleNet, ResNet50, ResNet101, and InceptionV3. The best-performing individual architecture in discriminating AD from CN and MCIc from CN is AlexNet, which scores 91% and 84% AUC for these two tasks, respectively. However, in discriminating MCIc from MCInc, the best individual model is ResNet101, with an AUC of 71%, presenting the same limitations of the ML systems in this task.

In such transfer-learning implementation, our main technical contribution was to propose an effective method to decompose 3D MRIs to RGB bands preserving the intra- and inter-subject spatial structural information of the original 3D MRI images, and to use these decompositions for transfer learning through efficient 2D pretrained CNN architectures. Specifically, in order to reduce the loss of information, different MRI-to-RGB decomposition approaches were implemented and then the combination of these different decomposition approaches was applied (see Table 2). The results obtained in this work on the 2D pre-trained architectures clearly show that the combination of different ways to decompose the MRI volumes into bi-dimensional images feeding pretrained architectures leads to higher performance in all diagnostic tasks. This may mean that different ways for decomposing MRI bring different effective information improving the discrimination power of systems trained on generic images for different AD phenotypes.

Spatial structural information is essential for 3D MRI images. Pre-trained 3D CNNs are emerging in the medical applications, however, 2D CNNs are still more common and have been more extensively used and validated in the literature. The advantage of using 2D pretrained architectures comes from the large availability of natural images used to pretrain such neural networks. If 2D pretrained architectures were applicable to 3D MRI volumes through our proposed decomposition followed by transfer learning, this would open new perspectives in the development of MRI-based transfer-learning models, not only for the diagnosis of AD, but also for other diseases (e.g. cancer, cardiovascular diseases) and other 3D medical images (e.g., CT, PET).

Regarding the architectures of the networks used for the proposed transfer-learning approach, pooling usually reduces the spatial size of the representation. However, this is quite mandatory in the learning process of deep architectures as it reduces the amount of information to be handled, which turns in a reduction of the computational costs. In our work, in order to preserve the as much as possible the spatial information, the maximum dimension of the adopted pooling filters was 3 by 3, thus warranting non-invasive reduction of spatial downsampling.

Our final transfer-learning strategy resulted in an ensemble of the different pre-trained architectures and in the combination of the different approaches for decomposing the MRI volumes and building *RGB-like* images that can be read by these architectures. This strategy was able to effectively discriminate AD-related phenotypes with the best accuracy of their independent transfer-learning architectures, resulting the following: 90% for AD vs. CN, 83% for MCIc vs. CN, and 71% for MCIc vs. MCInc.

The performance of our proposed transfer-learning methods (e.g., AUC in AD vs. CN 90% Method 2). is in line with the published papers on deep-learning techniques applied on many hundreds of MRI images for the diagnosis of AD and appeared since 2017. The majority of papers in this field of study focused on the discrimination of AD from CN. For example, Aderghal et al. (59) used a 2D-CNN approach by extracting information from the hippocampal ROI and further by applying data augmentation; their approach resulted in an accuracy of 91%. Cheng et al. (60) and Korolev et al. (61) adopted a 3D-CNN approach for the same task, the former reaching 92% AUC, the latter 88% AUC by implementing a ResNet-inspired architecture (42) It is worth noting that different strategies can be implemented extending beyond MRI images. For instance, Ortiz et al. (62) coupled MRI and PET imaging using an ensemble of deep-learning architectures on pre-defined brain regions, reaching 95% AUC for classifying AD vs. CN.

In a pioneering paper by Suk et al. (63), the authors trained on many hundreds of MRI images a 3D deep network with a restricted Boltzmann machine to classify MCIc vs. MCInc with 73% AUC (this paper 73% Method 1, 71% Method 2). Liu et al. (64) used a different strategy based on cascaded CNN pre-trained on 3D image patches to obtain 82.7% AUC for MCIc vs. CN when using several hundreds of MRI data (90.6% AUC for AD vs. CN), while Cui et al. (65) proposed an approach based on recurrent neural networks reaching 71.7% for MCIc vs. MCInc based on a set of hundreds longitudinal MRI images (91% accuracy for AD vs. CN).

From a computational point of view, our results show that an ensemble of 2D pre-trained CNNs is a promising approach, as it is able to reach performance that are comparable, although not significantly different, to that obtained by training from scratch 3D deep learning networks using many hundreds of medical images but with much less computational costs or limited number of available medical images. The fact that the performance of the proposed transfer-learning architectures, pre-trained on non-medical images, is similar to those of networks trained from scratch on medical images and using 3D spatial information, suggests that the features learned in pre-trained networks are effectively transferred to medical images with an important reduction in the number of images and, consequently, in the amount of computational time required for pre-training or training new deep networks from scratch.

To the best of our knowledge, this is one of the first studies performing a comparison of conventional ML, deep/transfer learning for the diagnosis and prognosis of AD on the same set of MRI volumes, thus obtaining comparable results. According to these results, the ensemble of 2D pre-trained CNNs showed comparable or slightly lower potential with respect to the fusion of conventional-ML systems. The 3D CNN trained from scratch on the original 3D MRI volumes from Salvatore-509 an Moradi-264 obtained lower performance than either the fusion of conventional-ML systems and the ensemble of 2D pre-trained CNNs, due to the limited sample of images used for training.

Focusing on the main comparison of the two approaches considered in this work –conventional ML vs. transfer learning-, the ensemble transfer-learning shows comparable results with the fusion of conventional-ML approach, even if the fusion of conventional ML is still higher than the ensemble transfer learning for the less complex tasks of AD vs. CN (93.2 vs. 90.2 % AUC) and MCIc vs. CN (90.6 vs. 83.2 % AUC). This better result obtained by the fusion of SVMs may be due to training from scratch.

Actually, there are advanced deep networks proposed recently for 3D MRI-based AD diagnosis and MCI-to-AD prediction of conversion, by using anatomical landmarks or dementia attention discovery schemes to locate those information in MRI brain regions, thus alleviating the small-sample-size problem (66–69). In our work we trained from scratch a new 3D CNN achieving lower performance with respect to the other considered 2D transfer learning methods (84% vs. [89-91]% for AD vs. CN, 72% vs. [80-84]% for MCIc vs. CN, 61% vs. [69-71]% for MCIc vs. MCInc). However, we would like to underline that training a 3D CNN from scratch requires a huge amount of training data. Although the estimation of the number of samples necessary to train a deep-learning classifier from scratch with good performance is still an open research problem, some studies tried to investigate this issue. According to Juba et al. (70) the amount of data needed for learning depends on the complexity of the model. A rule of thumb descending from their paper is that we need 10 cases per predictor to train a simple model like a regressor, while we need 1,000 images per class to train from scratch a deep-learning classifier like a CNN. These numbers are far from those available in our AD datasets, where the largest class in Salvatore-509 dataset was CN with 162 subjects, while the smaller was MCIc with 76 patients. This can also explain the lower performance of our 3D CNN with respect the published papers on deep-learning techniques applied on many hundreds of MRI images for the diagnosis of AD.

From a clinical point of view, this paper mainly focused on the whole-brain MRI volumes. This choice was made in order to preserve as much information associated to the early-AD disease as possible, which could come from different regions of the brain (e.g., parietal and posterior cingulate regions, precuneus, medial temporal regions, hippocampus, amygdala, and entorhinal cortex). The use of region-based biomarkers of given anatomical structures (e.g., hippocampus) would indeed risk excluding potentially-discriminant information for the early diagnosis of AD.

However, one of the analyses performed in this work focused on the inner cerebral structures (including hippocampus) instead of the entire MRI volume. From this point of view, this analysis returned an important feedback on the behavior of the trained models. As expected, models trained using the inner cerebral structures obtained higher performance than models based on whole-brain MRIs. Specifically, results show an effective improvement of classification performance for the most complex discrimination task of MCIc-vs-MCInc. The mean percentage AUC increases from 69.1 to 73.3. This uptrend is not replicated when considering the other comparisons (AD vs. CN, MCIc vs. CN) that remain stable. This behavior is not unexpected, because the inner cerebral structures (specifically, the medial temporal lobe including the parahippocampal gyrus) are known to be the first areas affected by the pathophysiological mechanisms of AD, even at prodromal or MCI stages (71). Because of this, selecting for the training data the inner cerebral-MRI structures can help the automatic classification of AD at early stages (MCIc vs. MCInc), at no costs on classification of diagnosis at later stages, when the pattern of atrophy is more widespread (e.g., 49). Even if not unexpected, these results show us the goodness of the learning process, including the preprocessing and the decomposition approach proposed in this work.

### Limitations

It must be noted that the results obtained by the proposed method may be influenced by two specific issues related to the application of transfer learning to MRI volumes. First and most important, deep-learning techniques -and CNNs in particular- are designed to best solve computer-vision tasks, such as visual object recognition or object detection (18). This makes them suitable for medical applications such as the automatic detection and segmentation of oncological lesions [e.g., (19, 72)], which typically involve locally-bounded regions. On the other side, the diagnosis of AD through the inspection of MRI scans involves the recognition of a pattern of cerebral atrophy that is distributed (not localized). Second, the use of transfer-learning techniques instead of training a new network from scratch may affect the learning stage of the network itself in the fine-tuning phase. As we mentioned above, the choice of using a transfer-learning approach was almost mandatory in this case, given the relatively low number of data available with respect to the number of samples needed to train a CNN model from scratch. However, the images used to pre-train the network (generic images) were particularly different from the images used to perform fine tuning and classification of new samples (MRI scans). This may reduce the potential of the network in learning feature representations that are specific of the medical-imaging context, thus affecting the final classification performance of the model. Notwithstanding these two criticisms, the results obtained in this paper are comparable with the state of the art and, thus, encourage the application of transfer learning to structural MRI data, even if further optimizations are required when considering networks pre-trained on generic images of different nature.

## Conclusions

In conclusion, in this paper we investigated the potential application of deep/transfer learning to the automatic early diagnosis and prognosis of AD compared to conventional ML.

The transfer-learning approach was able to effectively discriminate AD from CN with 90.2% AUC, MCIc from CN with 83.2% AUC, and MCIc from MCInc with 70.6% AUC, showing comparable or slightly lower results with respect to the fusion of conventional-ML systems (AD from CN with 93.1% AUC, MCIc from CN with 89.6% AUC, and MCIc from MCInc with AUC in the range of 69.1–73.3%). These results open new prospective on the use of transfer learning combined with neuroimages for the automatic early diagnosis and prognosis of AD, even if pretrained on generic images. A deep-learning network trained from scratch on few hundreds of MRI volumes obtained lower performance than either the fusion of conventional-ML systems and the ensemble of 2D pre-trained CNNs, due to the limited sample of images used for training.

## Data Availability Statement

Publicly available datasets were analyzed in this study. This data can be found here: ADNI (Alzheimer's Disease Neuroimaging Initiative).

## Author Contributions

LN, CS, and IC conceived of the present research work. IC was responsible for the retrieval of the datasets used in this study. LN, MI, and CS planned and implemented the data analyses (which refers to data preparation, image processing, and classification). LN, MI, and CS wrote the manuscript. SB, SP, RN, and IC reviewed and edited the manuscript. The work was supervised by IC and CS. All authors contributed to the article and approved the submitted version.

## Conflict of Interest

IC and MI own shares of DeepTrace Technologies S.R.L. CS is CEO of DeepTrace Technologies S.R.L. DeepTrace Technologies S.R.L is a spin-off of Scuola Universitaria Superiore IUSS Pavia, Italy. The remaining authors declare that the research was conducted in the absence of any commercial or financial relationships that could be construed as a potential conflict of interest.
