# Supplementary material for: Comparison of Transfer Learning and Conventional Machine Learning Applied to Structural Brain MRI for the Early Diagnosis and Prognosis of Alzheimer's Disease
Source: Front Neurol. 2020 Nov 5;11:576194. doi: 10.3389/fneur.2020.576194 (PMC7674838; doi:10.3389/fneur.2020.576194)
Supplement: Supplementary file 1 [file Table_1.docx]

Supplementary Material

# Supplementary Tables

**Table S1. Classification performance in terms of AUC of AlexNet and GoogleNet after fine tuning using different Gap values and MRI-decomposition approaches.** Results refer to the models that were fine tuned using the entire MRI volumes. The combination of different Gap values or *MRI-decomposition* approaches is simply referred to as *combination*.

|  |  |  | *Salvatore-509*  *dataset* | *Salvatore-509*  *dataset* | *Salvatore-509*  *dataset* | *Moradi-264*  *dataset* |
| --- | --- | --- | --- | --- | --- | --- |
| Pretrained architecture | Gap | MRI-decomposition approach | AD vs CN | MCIc vs CN | MCIc vs MCInc | MCIc vs MCInc |
| AlexNet | 0 | A | 88.2 | 81.9 | 61.8 | 69.2 |
|  | 0 | B | 87.0 | 79.8 | 62.5 | 65.1 |
|  | 0 | C | 89.6 | 74.7 | 63.8 | 59.6 |
|  | 0 | D | 90.1 | 82.2 | 65.5 | 68.7 |
|  | 0 | A, B, C, D (combination) | 90.4 | 83.2 | 65.8 | 68.2 |
|  | 0, 1, 2 (combination) | A, B, C, D (combination) | 90.8 | 84.2 | 68.2 | 70.0 |
| GoogleNet | 0 | A | 86.4 | 80.2 | 66.5 | 65.2 |
|  | 0 | B | 87.2 | 75.0 | 68.9 | 68.9 |
|  | 0 | C | 86.3 | 78.0 | 68.7 | 68.4 |
|  | 0 | D | 87.5 | 79.6 | 65.2 | 68.9 |
|  | 0 | A, B, C, D (combination) | 88.6 | 80.2 | 70.5 | 69.7 |
|  | 0, 1, 2 (combination) | A, B, C, D (combination) | 89.6 | 81.6 | 70.9 | 69.1 |

**Table S2. Classification performance in terms of AUC (std, if present) of the different deep/transfer-learning pretrained architectures considered individually.**

|  | *Salvatore-509*  *dataset* | *Salvatore-509*  *dataset* | *Salvatore-509*  *dataset* | *Moradi-264*  *dataset* |
| --- | --- | --- | --- | --- |
| Architecture | AD vs CN | MCIc vs CN | MCIc vs MCInc | MCIc vs MCInc |
| AlexNet^P^ | 90.79 | 84.21 | 68.19 | 70.02 |
| GoogleNet^P^ | 89.56 | 81.61 | 70.88 | 69.07 |
| ResNet50^P^ | 89.75 | 81.83 | 69.66 | 71.05 |
| ResNet101^P^ | 89.86 | 82.24 | 70.40 | 72.07 |
| InceptionV3^P^ | 88.76 | 79.89 | 67.34 | 72.29 |
| 3D CNN | 84.14 | 72.25 | 60.09 | 62.09 |

*For pretrained 2D architectures (entries from 1 to 5), results refer to the ensable of models trained using different Gap values (0, 1, 2) and MRI-decomposition approaches (A, B, C, D) on the entire MRI volumes.*

*P, pretrained.*

**Table S3. Classification performance (AUC) of conventional ML (entries 1-3) and ensemble transfer learning (entry 4).** Results refer to the conventional-ML models trained using different feature-extraction and classification techniques as well as to the ensemble of 5 transfer-learning models. The entire MRI volumes were used.

|  | *Salvatore-509*  *dataset* | *Salvatore-509*  *dataset* | *Salvatore-509*  *dataset* | *Moradi-264*  *dataset* |
| --- | --- | --- | --- | --- |
|  | AD vs CN | MCIc vs CN | MCIc vs MCInc | MCIc vs MCInc |
| AS + SVM | 93.1 | 89.6 | 64.6 | 73.6 |
| kPLS+ SVM | 93.3 | 90.8 | 63.5 | 67.8 |
| Fusion of 2 SVMs | 93.2 | 90.6 | 64.2 | 73.9 |
| Ensemble of 5 transfer-learning models | 90.2 | 83.2 | 70.5 | 70.6 |

**Table S4. Classification performance (AUC) of conventional ML (entries 1-3) and ensemble transfer learning (entry 4).** Results refer to the conventional-ML models trained using different feature-extraction and classification techniques as well as to the ensemble of 5 transfer-learning models. The entire MRI volumes were used. The inner cerebral structures (including the hippocampal region) from each MRI scan were used.

|  | *Salvatore-509*  *dataset* | *Salvatore-509*  *dataset* | *Salvatore-509*  *dataset* | *Moradi-264*  *dataset* |
| --- | --- | --- | --- | --- |
|  | AD vs CN | MCIc vs CN | MCIc vs MCInc | MCIc vs MCInc |
| AS + SVM | 93.0 | 89.2 | 73.5 | 73.5 |
| kPLS+ SVM | 93.2 | 90.0 | 68.2 | 67.9 |
| Fusion of 2 SVMs | 93.0 | 90.4 | 72.8 | 73.8 |
| Ensemble of 5 transfer-learning models | 90.4 | 83.0 | 70.3 | 70.8 |
